# The Infection Rate of Bird-Feeding *Ixodes ricinus* Ticks with *Borrelia garinii* and *B. valaisiana* Varies with Host Haemosporidian Infection Status

**DOI:** 10.3390/microorganisms11010060

**Published:** 2022-12-25

**Authors:** Alžbeta Šujanová, Zuzana Čužiová, Radovan Václav

**Affiliations:** Institute of Zoology, Slovak Academy of Sciences, Dúbravská Cesta 9, 84506 Bratislava, Slovakia

**Keywords:** *Ixodes ricinus*, *Borrelia*, Haemosporida, avian hosts, seasonality, host age

## Abstract

Background: Birds are known to maintain and spread human pathogenic borreliae, but they are common hosts of diverse parasite communities, notably haemosporidians. Only a few studies examined whether tick infestation and/or *Borrelia* prevalences vary with hosts’ haemosporidian infection status. Methods: Here, we study whether *Ixodes ricinus* infestation rates and *Borrelia* infection rates in bird-feeding ticks vary according to haemosporidian infection status in a community of free-living avian tick hosts. Results: Birds of six avian species harbored the majority of ticks. Both the tick infestation prevalence and the intensity peaked during spring and summer, but while bird-feeding nymphs prevailed in spring, bird-feeding larvae dominated in summer. Almost half of the bird-feeding ticks were found to be positive for *B. burgdorferi* s.l. Although the majority of infections involved bird-associated *B. garinii* and *B. valaisiana*, *B. garinii* appears to be the dominant *Borrelia* strain circulating in locally breeding avian species. We detected a negative link between the hosts’ haemosporidian infection status and the *Borrelia* infection rate of bird-feeding ticks, but the association was dependent on the host’s age. Conclusions: Our results on tick infestation intensity support the idea that more immunologically vulnerable hosts harbor more ticks but suggest that different mechanisms may be responsible for tick infestation rates among immunologically naïve and experienced avian hosts. The results on *Borrelia* infection rates in bird-feeding ticks are consistent with studies revealing that intracellular parasites, such as haemosporidians, can benefit from the host immune system prioritizing immune responses against extracellular parasites at the expense of immune responses against intracellular parasites. The findings of our study urge for a more robust design of parasitological studies to understand the ecology of interactions among hosts and their parasites.

## 1. Introduction

Spirochetes of the *Borrelia burgdorferi* sensu lato (s.l.) complex are diverse and widespread pathogens vectored by ticks of the *Ixodes ricinus* complex [1]. Birds are common hosts of ixodid ticks, playing an important role in the ecology of tick-borne pathogens [2]. In addition, birds are known to maintain and spread, among others, *B. garinii* [3,4], the causative agent of Lyme neuroborreliosis in humans [5]. Importantly, birds are common hosts of diverse parasite communities, including haemosporidians [6]. Even though different parasites routinely covary within hosts [7], only a few studies examined whether tick infestation and/or *Borrelia* prevalences vary with the hosts’ haemosporidian infection status [8,9,10].

Tick loads vary widely among avian hosts but were found to scale with host body size or condition [10,11], age [8,12] and host foraging behavior [11,13,14,15]. In an experimental study, ticks were found to aggregate more on physically well-developed but less-immunocompetent nestlings [16], consistent with the idea that birds providing higher nutritional value and posing lower risks of host immune responses represent prime hosts for hematophagous arthropods [17,18,19]. Intriguingly, host attributes may not only determine tick loads, but also the probability of infestation with parasite-infected ticks. This is because tick infection with certain parasites, such as *Borrelia* spirochetes, can “manipulate” the behavior of questing *I. ricinus* ticks and increase their host-seeking efficacy [20,21,22,23].

Tick loads as well as the prevalences of *Borrelia* and apicomplexan parasites show temporal dynamics within seasons, and covariation between these parasites indeed can change over time [24]. Previously, we reported for Slovakia that while the number of questing ticks and *B. garinii* prevalence in questing and bird-feeding ticks peak in spring and summer, autumn is characterized by low numbers of questing ticks and higher prevalences of *B. afzelii* and *B. valaisiana* [25,26]. Similarly, we showed for the same study site that the pattern of host infections with avian haemosporidians changes within seasons with respect to infection prevalence and intensity [27]. A feature shared by both *Borrelia* and haemosporidian parasites, these parasites can persist in hosts across different seasons in various immune-privileged sites following the infectious period in the skin and blood [6,28,29,30,31]. Likewise, relapses in both avian borreliosis and hemosporidiosis occur in spring in European birds, presumably due to hormonal and immunological responses to migration [32,33]. These results, but also the results of other studies [9], suggest that while borreliae and apicomplexans routinely coinfect their hosts, the interactions between ticks, parasites and hosts can vary temporally [24].

Even though ticks, borreliae and haemosporidians are common avian parasites, covariations between these parasites did not receive much attention. The results of previous studies did not show any association between tick and/or *Borrelia* prevalences with respect to hosts’ haemosporidian infection status [8,9,10]. Although these results may indicate that the parasites occur independently within hosts, one of the latter studies detected a tendency for a higher probability of haemosporidian infection in birds infected with borreliae [9]. In contrast to the above studies, multiple studies have demonstrated that intracellular apicomplexan and intracellular bacterial parasites routinely covary. In an experimental study, avian malaria parasitaemia was found to be higher in birds coinfected with the bacterium *Mycoplasma gallisepticum* [34]. Similarly, multiple studies detected positive associations between extracellular *Borrelia* spp. and intracellular *Babesia* spp. within hosts [24,35,36]. Analogously to the studies on extracellular bacterial parasites, multiple studies found a positive role of extracellular intestinal parasites in the prevalence and parasitaemia of different apicomplexans [37,38,39,40,41].

The aim of this study is to determine whether tick infestation and *Borrelia* infection in bird-feeding ticks vary according to haemosporidian infection status in free-living avian tick hosts. We consider two scenarios to explain the variation in *Borrelia* prevalences in bird-feeding *I. ricinus* ticks and in tick infestation prevalence and intensity. In both scenarios, ticks should aggregate more on hosts with lower immune defense capacities, as suggested by works on avian blood-feeding arthropods [16,18,19]. Since increased blood parasitaemia is thought to reflect decreased immune defense capacities in avian hosts [42], we predict that more ticks should be found on haemosporidian-positive birds. Importantly, tick aggregation on specific hosts could be enhanced by *Borrelia* infection in questing ticks, as *Borrelia* infection can boost tick host-seeking efficacy [20,21,22,23]. In the tick-centered scenario, therefore, haemosporidian-positive compared to haemosporidian-negative avian hosts should harbor more ticks with respect to both absolute and relative numbers of *Borrelia*-infected ticks. Alternatively, in the host-centered scenario, variation in the *Borrelia*-infection rates of bird-feeding ticks could mainly reflect variation in host-to-tick *Borrelia* transmission rates, with hemosporidiosis being a manifestation of late host immune responses to extracellular parasites, such as *Borrelia* spirochetes. In this scenario, given hemosporidiosis arises from the host suppression of extracellular bacteria at the expense of immunologically conflicting responses to intracellular parasites [24,43,44], *Borrelia*-infection rates should be lower in ticks feeding on haemosporidian-positive birds.

## 2. Materials and Methods

### 2.1. Study Area, Field Methods and Study Species

Ticks and birds were sampled in Slovakia at the Drienovec Bird Ringing Station (48°36′58.7″ N; 20°54′53.6″ E) as a part of research on vector-borne avian parasites [26,27,45]. The study site (ca. 7.7 ha) is represented by a mosaic of woody wetland and forest-meadow ecotones at 190 m a.s.l. Birds were captured with mist nests and banded under the permit of the Ministry of the Environment of the Slovak Republic No. 269/132/05-5.1_pil and 9830/2017-6.3. Bird capture took place for 3 years (2017, 2018 and 2019) between April and November. Sampled birds were carefully examined for attached ticks, which were removed with fine forceps in tubes with 70% ethanol. Ticks for each bird were stored in separate tubes. The blood sample was taken from a brachial vein and ring code, species and, if possible, age and sex were recorded for each bird sampled, and the birds were subsequently released. Blood samples were stored in 70% ethanol at 4 °C until DNA extraction (within 1–7 months). Birds were sampled each year in the second half of April, between mid-June and mid-July and between mid-September and the beginning of November. These three sampling periods were chosen to obtain representative samples of avian parasites during the spring migration, breeding and autumn migration periods, respectively. Ticks were identified by species and life stages using available taxonomic keys [46,47]. *Borrelia* prevalence was examined for *I. ricinus* ticks as this species is the principal vector of borreliae in Europe [48].

### 2.2. DNA Extraction

The DNA from individual *I. ricinus* ticks was extracted by the alkaline-hydrolysis method [49] and eluted in 125 (larvae) and 250 (nymphs and adults) µL of MilliQ water [50]. DNA samples were stored at −20 °C. A 620-bp fragment of tick mitochondrial gene cytochrome *b* (*cyt b*) was amplified in each extracted sample to confirm the presence of tick DNA [51]. Only positive samples were further analyzed for the presence of tick-borne agents.

DNA extractions for avian blood samples collected in 2017 and 2019 were performed using the QIAamp DNA Blood Kit (Qiagen, Hilden, Germany) following the manufacturer’s recommendation. DNA from samples obtained in 2018 was extracted using a standard phenol-chloroform extraction with ethanol precipitation [52]. Extracted DNA was resolved to a final concentration of ca. 100 ng/μL and stored at −20 °C until subsequent analyses. The quantity and quality of DNA samples was assessed by NanoPhotometer Pearl (Implen, Munich, Germany).

### 2.3. Borrelia Molecular Analyses

Each tick was tested for the presence of *B. burgdorferi* s.l. DNA. PCR amplification of a 222–255-bp fragment of rrfA-rrlB intergenic spacer was carried out using primers IGSA 5′-CGACCTTCTTCGCCTTAAAGC-3′ and IGSB 5′-AGCTCTTATTCGCTGATGGTA-3′ [53]. The PCR reaction was performed in a total reaction mixture of 25 µL. The PCR reaction mixture per each sample contained 2.5 µL of 10× PCR buffer, 1 µL of 25 mM MgCl_2_, 0.125 µL of HotStartTaq DNA polymerase (Qiagen, Hilden, Germany), 0.5 µL of both IGSA and IGSB primers (10 µM), 0.5 µL 10 mM dNTP (Thermofisher, Dreieich, Germany) and 14.875 µL of nuclease-free water (Promega, Madison, WI, USA). A quantity of 5 µL of tick DNA template was added to the reaction mixture. A touch-down PCR program was set following Derdáková et al. [53]. Positive samples were typed into borrelial genospecies by restriction fragment length polymorphism (RFLP) analysis following Derdáková et al. [53]. A quantity of 13 µL of PCR product was mixed with 0.5 µL of Tru1I restriction enzyme (Fermentas, Thermo Scientific, Vilnius, Lithuainia) and 1.5 µL of buffer (Fermentas, Thermo Scientific, Vilnius, Lithuania). Digestion ran at 65 °C for 5 min. Electrophoretic separation was performed in the Origins system (Elchrom Scientific, Cham, Switzerland) using Spreadex EL300 mini gel (Elchrom Scientific, Cham, Switzerland) at 120 V for 150 min. After electrophoresis, the gel was stained with SYBR green (Sigma-Aldrich, St. Louis, MO, USA) for 45 min and visualized by UV transilluminator (Vilber-Lourmant; Sigma-Aldrich, St. Louis, MO, USA).

### 2.4. Avian Haemosporidian Molecular Analyses

The DNA samples were examined for avian haemosporidian infection using quantitative real-time PCR (qPCR) targeting 182 bp fragment of *cyt b* gene [54]. All reactions were carried out using GoTaq qPCR Master Mix (Promega, Madison, WI, USA) on a CFX96 real-time thermocycler (Bio-Rad, Hercules, CA, USA). The total volume of the reactions was 20 μL, containing 10 μL of GoTaq qPCR Master Mix 2×, 0.5 μL of each primer (10 μM concentration), 6 μL of molecular grade water and 3 μL of DNA template (ca. 300 ng). The following cycling conditions were used: 95 °C for 2 min, followed by 40 cycles of 95 °C for 30 s and 64 °C for 35 s (with a plate read) followed by a final melt curve analysis using instrument default settings. The samples were run in duplicate for all of the samples, together with two non-template controls to check for non-specific amplifications. At the end of the reactions, the amplification curves and melting curves were inspected to obtain values of threshold cycles (Ct) for each sample and determine false positives [55]. For quantification of parasites, seven samples with known quantities [54] were included in each reaction to establish amplification efficiencies and standard curves. A synthetic double-stranded DNA product (Eurofins Genomics, Ebersberg, Germany), designed from a 220 bp fragment of the conserved rDNA region of *Plasmodium relictum*, accession #NC012426 [54], was used as a positive control. The DNA was diluted to a starting concentration of 106 copies/μL using online calculator on www.thermofisher.com (accessed on 1 May 2021). This starting solution was then serially diluted by 10-fold to prepare a series of solutions from 106 copies of genomic DNA per μL down to 1 copy/μL (that is, there were 7 dilutions: 106, 105, 104, 103, 102, 101 and 100). To determine the microarray limit of detection, 3 μL of these diluted DNA samples were used as templates for the amplifications of the *cyt b* gene. Details on criteria for accepting qPCR data as valid and results on the population structure of avian haemosporidian can be found in Šujanová et al. [27].

### 2.5. Statistical Analysis

Contingency tables were examined with the χ2 and Fisher exact tests to determine the discrepancies between observed and expected frequencies in tick prevalences. The Bayesian framework was used to examine variations in tick infestation prevalence, tick infestation intensity, *Borrelia* prevalence and the rate of *Borrelia* infection in bird-feeding ticks. The Bayesian approach was used because it offers power and flexibility for complex models, such as those with nested and heterogenous count data [56]. In the majority of models, we used the same fixed (population-level) and random (group-level) parameters. Specifically, the hosts’ haemosporidian infection status (binary categorical variable), time of year (categorical variable with three levels: spring, summer and autumn) and the interaction between the two variables were used as population-level parameters. In addition, age was considered with the latter two parameters in a full factorial design for a subset of birds with known ages to examine variations in tick infestation intensity and the *Borrelia* infection rate of bird-feeding ticks. In turn, year (categorical variable with 3 levels) and host species (categorical variable with 4–6 levels corresponding to avian host species), which was nested within year, were used as group-level parameters throughout analyses (Tables S1–S4). In two models for subsets of four bird hosts, nesting of host species within year was not used due to sample size limitations for some species–year categories, whereby year and host species were used as group-level parameters (Tables S5–S6). We do not employ more complex random structures (e.g., nesting host species within year and time of year), because such models turned out with specification problems and had inferior predictive performance. In addition, tick life stage is not included in models as a population- or group-level parameter, because of the lack of robustness of such models.

Tick infestation and *Borrelia* infection prevalences were examined assuming the Bernoulli error distribution with the logit link. Tick infestation intensity and *Borrelia*-infection rates of bird-feeding ticks were examined with zero inflated negative binomial models with the log link. Negative binomial models were used instead of Poisson models to account for overdispersion of count data. In addition, zero inflation was employed in the negative binomial models as this extension improved the model fit over ordinary negative binomial distribution models. The rate of *Borrelia*-infected bird-feeding ticks per host was examined as the number of *Borrelia*-positive ticks per host while host tick infestation intensity was used as the offset parameter. Model specifications, along with parameter estimates and corresponding statistics, are detailed in Tables S1–S6.

Predictors of tick infestation intensity (Tables S1–S3) were studied for six avian species that carried the majority of ticks sampled: the Eurasian blackbird *Turdus merula*, song thrush *Turdus philomelos*, European robin *Erithacus rubecula*, hawfinch *C. coccothraustes*, great tit *Parus major* and Eurasian blackcap *Sylvia atricapilla*. Predictors of *Borrelia* infection prevalence (Table S4) were studied for five avian species that carried at least one *Borrelia* infected tick: the Eurasian blackbird, song thrush, European robin, hawfinch and great tit. Finally, predictors of the *Borrelia* infection rate (Tables S5–S6) were studied for four avian species that represented the locally most frequent hosts of *Borrelia* infected ticks: the Eurasian blackbird, song thrush, European robin and great tit. As for the latter response variable, only birds that carried at least one tick were included in analyses.

For population-level parameters, we used weakly informative priors through the brms package [57]. Parameter estimation was conducted with the brms package using the Markov chain Monte Carlo (MCMC)-based static Hamiltonian Monte Carlo and no-U-turn sampler algorithms through the Stan platform [57,58]. Each model was fitted using 4 independent Markov chains, each with 20,000 iterations and 500 warm-up samples.

Chain trace plots and the $\hat{R}$ statistic for each parameter were explored to check for convergence, while the effective sample size (ESS) statistics were used to check for the reliability of posterior means and variances of parameter estimates [59]. The $\hat{R}$ statistic was <1.01 for all parameters across models (Tables S1–S6). A qualitative posterior predictive check was conducted visually through plots to verify that the model reasonably mimicked the data. In addition, the Pareto *k* diagnostics obtained through leave-one-out cross validation (LOO) was used to check the reliability of estimates [60]. The Pareto *k* values were lower than 0.7 for >99.3% of observations across models.

Each model parameter is summarized using the mean of the posterior distribution and 95% quantile-based credible intervals (CrI; Tables S1–S6). Bayesian inference was based on posterior probability hypothesis testing through the brms package [57]. Specifically, we tested one-sided hypotheses on the differences between the levels of population-level factors, whereby the hypotheses were considered to be strongly supported by the data when the posterior probability exceeded 95%. Posterior estimates and 90% CrIs for one-sided hypotheses are reported on the response scale by back-transforming parameter estimates from the logit or log scales.

All analyses were conducted with R software 4.0.2 [61]. Bayesian models were constructed using the brms package [57]. Plots were constructed using the ggplot2 [62] and tidybayes [63] packages.

## 3. Results

### 3.1. General Tick Infestation and Borrelia Infection Patterns

We examined 1852 birds of 61 species for *I. ricinus* infestation and *B. burgdorferi* s.l. infection status of bird-feeding ticks. Bird-feeding *I. ricinus* ticks (*n* = 1653) were collected from 18% (*n* = 340) of birds of 20 species (Table 1). In addition to *I. ricinus* ticks, ten (4 larvae/6 nymphs) *Haemaphysalis concinna* bird-feeding ticks were collected. Birds of as few as 6 avian species (Eurasian blackbird, song thrush, European robin, hawfinch, great tit and Eurasian blackcap) harbored the majority (*n* = 1570; 95%) of the bird-feeding *I. ricinus* ticks (Table 1). Only two avian species, Eurasian blackbird and song thrush, were regularly infested with ticks (Table 1). Tick infestation intensity varied markedly among the 6 avian species (Eurasian blackbird: 1–51 ticks; song thrush: 1–42; European robin: 1–12; hawfinch: 1–11; great tit: 1–11 and Eurasian blackcap: 1–5, Figure S1).

Of the bird-feeding *I. ricinus* ticks, 764 ticks (46%) were infected with *B. burgdorferi* s. l., with the majority (724; 95%) of infected ticks involving *B. garinii* and *B. valaisiana* (Table 1). Most bird-feeding tick larvae were collected during summer, but the infection rate between infected and uninfected larvae differed from spring to autumn; infected larvae showed higher-than-expected numbers in summer, but lower-than-expected numbers in autumn (spring/summer/autumn: *Borrelia*-negative larvae—58/232/131, *Borrelia*-positive larvae—25/310/39; χ^2^ = 71.61, df = 2, *p* < 0.001). As for nymphs, they were collected from birds mostly during spring, but the link between infection status and time of year was not significant (spring/summer/autumn: *Borrelia*-negative nymphs—292/89/87, *Borrelia*-positive nymphs—245/91/54; χ^2^ = 4.81, df = 2, *p* = 0.09).

The ratio of infected larvae to nymphs was markedly higher for tick infections involving *B. garinii* than *B. valaisiana* (larvae/nymphs infected: *B. garinii*—312/213, *B. valaisiana*—94/199; Fisher exact test, *p* < 0.001). While there was a common trend for larval infection with both *B. garinii* and *B. valaisiana* to peak during summer, the link between *Borrelia* genospecies and the time of year was significant due to a shallower temporal trend for *B. valaisiana* infections (spring/summer/autumn: *B. garinii*—12/279/21, *B. valaisiana*—16/55/23; χ^2^ = 47.54, df = 2, *p* < 0.001). In addition, while nymphal infections with both *B. garinii* and *B. valaisiana* peaked during spring, temporal trends differed between the 2 *Borrelia* genospecies due to a higher-than-expected infection rate of *B. garinii* during summer (spring/summer/autumn: *B. garinii*—119/68/26, *B. valaisiana*—138/29/32; χ^2^ = 17.25, df = 2, *p* < 0.001).

### 3.2. General Haemosporidian Infection Patterns

Blood samples of 1851 birds were examined for haemosporidian infection, with 767 of 1775 (43%) birds of 61 species testing unambiguously positive for avian haemosporidians. The avian species showing the highest haemosporidian prevalence largely overlapped with the 6 species harboring most of the *I. ricinus* ticks collected (haemosporidian prevalence—Eurasian blackbird: 67%; song thrush: 78%; European robin: 25%; hawfinch: 60%; great tit: 53% and Eurasian blackcap: 60%). We were able to determine haemosporidian infection status for 1040 of 1081 (96%) birds of these 6 species.

### 3.3. Association between Tick Infestation and Host Haemosporidian Infection Status

**Figure 1:**
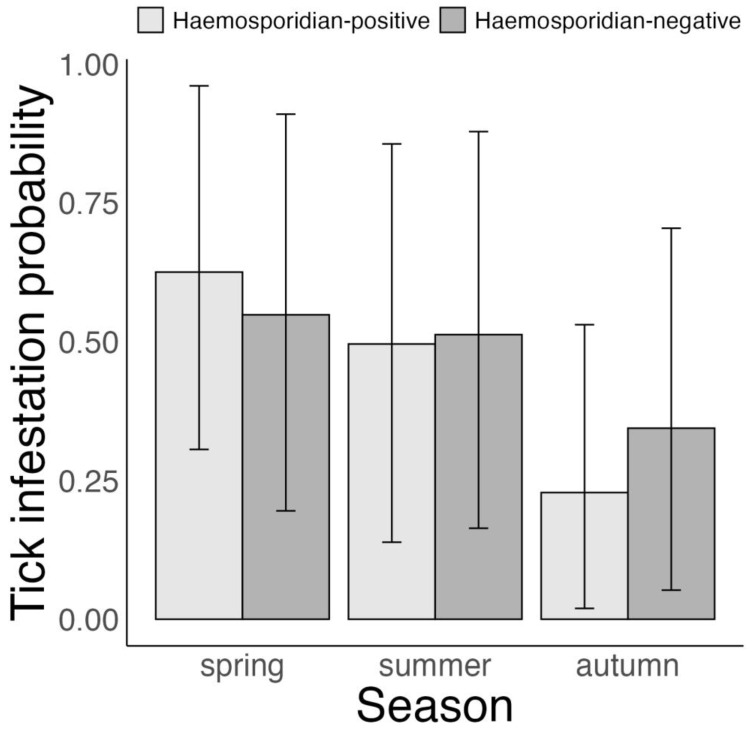
Bayesian Bernoulli distribution model on variation in *Ixodes ricinus* infestation probability according to haemosporidian infection status of 6 avian host species in Slovakia, 2017–2019. Displayed are posterior means and 95% CrI.

The probability of infestation with *I. ricinus* ticks in the 6 avian species was on average 40 and 29% lower in autumn than spring and summer, respectively (posterior mean estimate (90% CrI), posterior probability for one-sided hypotheses—autumn vs. spring: –40% (–54 to –23%), *p* = 1; autumn vs. summer: –29% (–43 to –14%), *p* = 1 and spring vs. summer: 11% (0 to 26%), *p* = 0.95, Figure 1, Table S1). In addition, the probability of tick infestation was linked to the hosts’ haemosporidian infection status, but only during autumn (haemosporidian-positive vs. haemosporidian-negative hosts—spring: 8% (–6 to 22%), *p* = 0.83; summer: –2% (–14 to 1%), *p* = 0.41 and autumn: –12% (–23 to –2%), *p* = 0.99, Figure 1).

**Figure 2:**
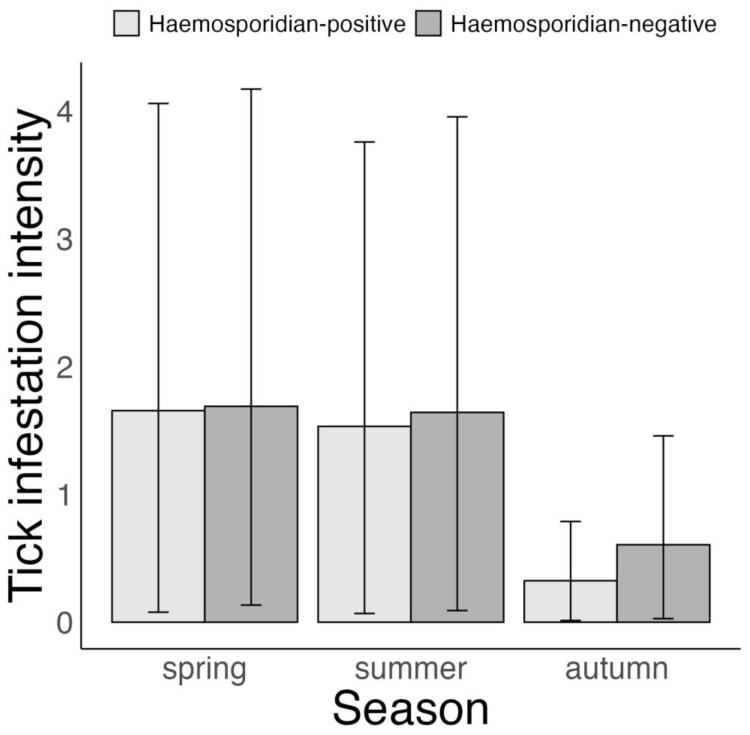
Bayesian zero-inflated negative binomial model on variation in *Ixodes ricinus* infestation intensity according to haemosporidian infection status of 6 avian host species in Slovakia, 2017–2019. Displayed are posterior means and 95% CrI.

Tick infestation intensity had a similar pattern to tick infestation probability with respect to the time of year and the hosts’ haemosporidian infection status. Specifically, tick infestation intensity in autumn was on average 1.36 and 1.2 ticks lower than that in spring and summer (autumn vs. spring: –1.36 ticks (–0.29 to –3.38 ticks), *p* = 1; autumn vs. summer: –1.2 ticks (–0.25 to –2.98 ticks), *p* = 1 and spring vs. summer: 0.16 ticks (–0.47 to 0.96 ticks), *p* = 0.66, Figure 2, Table S2). Additionally, tick infestation intensity was linked to the hosts’ haemosporidian infection status, but only during autumn (haemosporidian-positive vs. haemosporidian-negative hosts—spring: 0 ticks (–0.74 to 0.76 ticks), *p* = 0.49; summer: –0.1 ticks (–0.77 to 0.5 ticks), *p* = 0.63 and autumn: –0.29 ticks (–0.77 to –0.04 ticks), *p* = 1, Figure 2).

**Figure 3:**
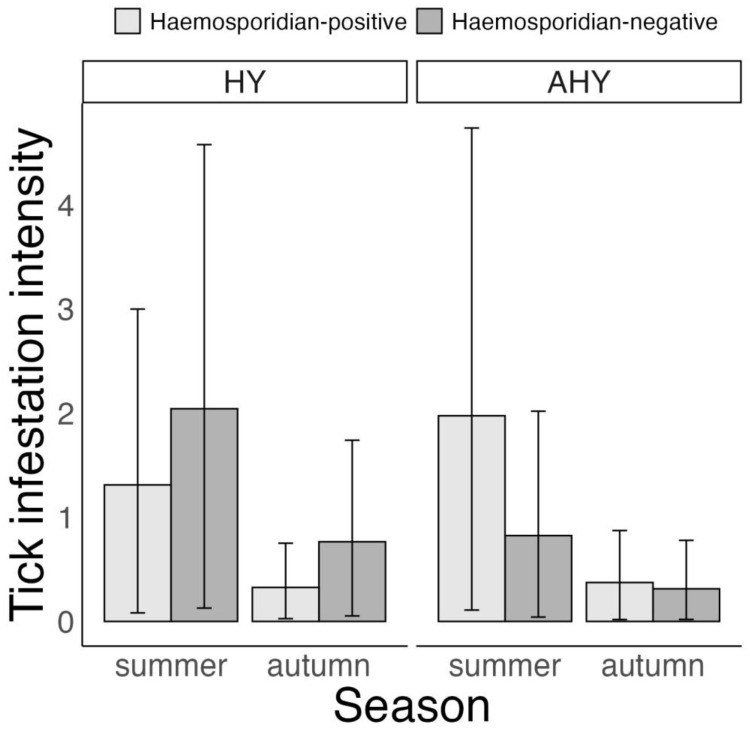
Bayesian zero inflated negative binomial model on variation in *Ixodes ricinus* tick infestation intensity according to haemosporidian infection status and age of six avian host species in Slovakia, 2017–2019. Displayed are posterior means and 95% CrI. Host age was classified as hatch-year (HY) and after-hatch-year (AHY). Note that spring was not considered in the model because only AHY birds were captured at that time of year.

For the summer–autumn subset of birds of known age, tick infestation intensity was found to be associated with the hosts’ haemosporidian infection status, host age and time of year. Namely, tick infestation intensity was higher for haemosporidian-positive than haemosporidian-negative birds in after-hatch-year (AHY) birds, but only in summer (haemosporidian-positive vs. haemosporidian-negative hosts—AHY birds, summer: 1.15 ticks (0.06 to 3.2 ticks), *p* = 0.96 and autumn: 0.06 ticks (–0.21 to 0.37 ticks), *p* = 0.34). In turn, infestation intensity was higher for haemosporidian-negative than haemosporidian-positive birds in hatch-year (HY) birds, but only in autumn (haemosporidian-positive vs. haemosporidian-negative hosts—HY birds, summer: –0.73 ticks (–2 to 0.01 ticks), *p* = 0.95 and autumn: –0.44 ticks (–1.06 to –0.09 ticks), *p* = 1, Figure 3, Table S3). Finally, while there was a trend for HY birds to have higher infestation intensity than AHY birds, the probability of this difference exceeded 95% only for autumn (HY vs. AHY, summer: 0.55 ticks (–0.84 to 2.27 ticks), *p* = 0.77 and autumn: 0.4 ticks (0.02 to 1.08 ticks), *p* = 0.96).

### 3.4. Association between Tick Borrelia Infection and Host Haemosporidian Infection Status

**Figure 4:**
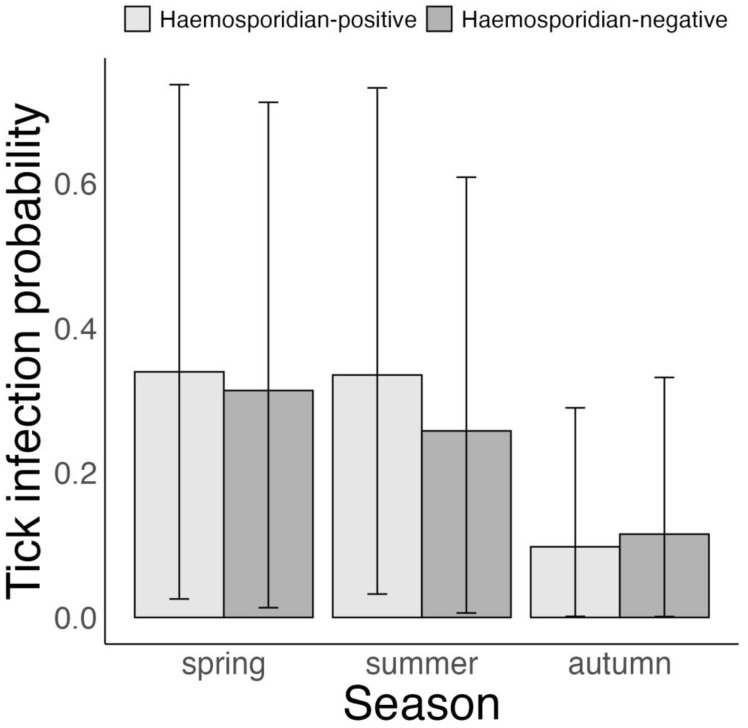
Bayesian Bernoulli distribution model on variation in the probability of *Borrelia*-infection of bird-feeding *Ixodes ricinus* ticks per host according to haemosporidian infection status of 5 avian host species in Slovakia, 2017–2019. Displayed are posterior means and 95% CrI.

The probability that an avian host carries at least 1 bird-feeding tick infected with *B. garinii* and/or *B. valaisiana* was on average 27 and 26% higher in spring and summer than in autumn (autumn vs. spring: –27% (–52 to –5%), *p* = 1; autumn vs. summer: –26% (–51 to –5%), *p* = 1 and spring vs. summer: 0% (–18 to 19%), *p* = 0.52, Figure 4, Table S4). However, *Borrelia* infection probability in bird-feeding ticks was not linked to the hosts’ haemosporidian infection status (haemosporidian-positive vs. haemosporidian-negative hosts—spring: 3% (–19 to 25%), *p* = 0.59; summer: 8% (–7 to 25%), *p* = 0.8 and autumn: –2% (–11 to 6%), *p* = 0.64, Figure 4).

**Figure 5:**
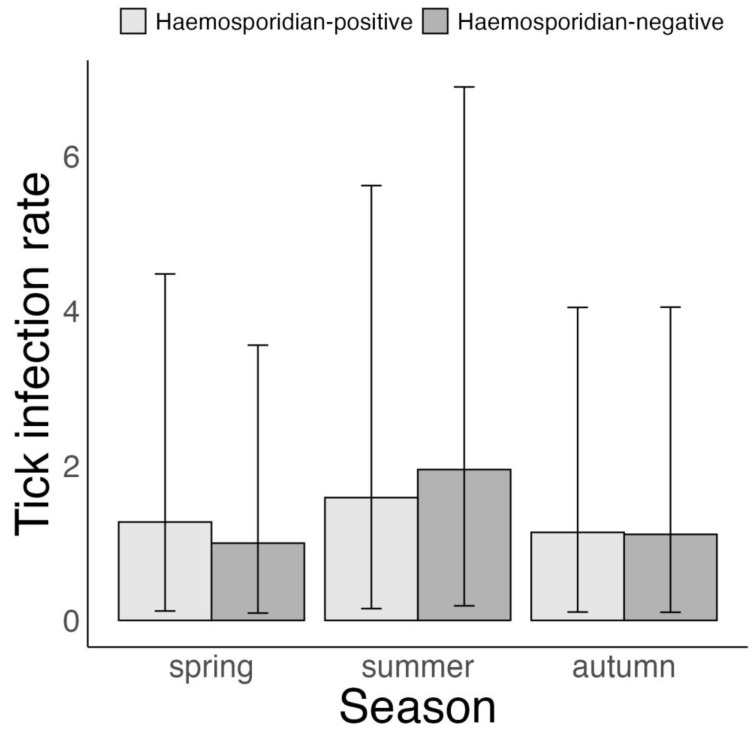
Bayesian zero inflated negative binomial model on variation in the rate of *Borrelia*-infected bird-feeding *Ixodes ricinus* ticks per host according to haemosporidian infection status of 4 avian host species in Slovakia, 2017–2019. The plotted rates are shown relative to the average tick infestation of 5.5 ticks per host. Displayed are posterior means and 95% CrI.

Relative to the average host infestation intensity (5.51 ticks), the *Borrelia* infection rate of bird-feeding ticks was on average 0.32 and 0.47 ticks higher in summer than in spring and autumn, respectively (autumn vs. spring: –0.15 ticks (–0.64 to 0.13), *p* = 0.76; autumn vs. summer: –0.47 ticks (–1.35 to –0.03), *p* = 0.99 and spring vs. summer: –0.32 ticks (–0.92 to –0.01), *p* = 0.97, Figure 5). Importantly, the tick infection rate was linked to the hosts’ haemosporidian infection status (Figure 5 and Table S5). Specifically, while in spring the tick infection rate was on average 0.27 ticks higher in haemosporidian-positive than haemosporidian-negative hosts (haemosporidian-positive vs. haemosporidian-negative hosts: 0.27 ticks (0 to 0.82), *p* = 0.95), in summer the difference was larger (0.37 ticks) but reversed (haemosporidian-positive vs. haemosporidian-negative hosts: –0.37 (–1.11 to 0), *p* = 0.95). In autumn, the hosts’ haemosporidian infection status was not associated with the tick infection rate (haemosporidian-positive vs. haemosporidian-negative hosts: 0.03 ticks (–0.49 to 0.57), *p* = 0.46).

**Figure 6:**
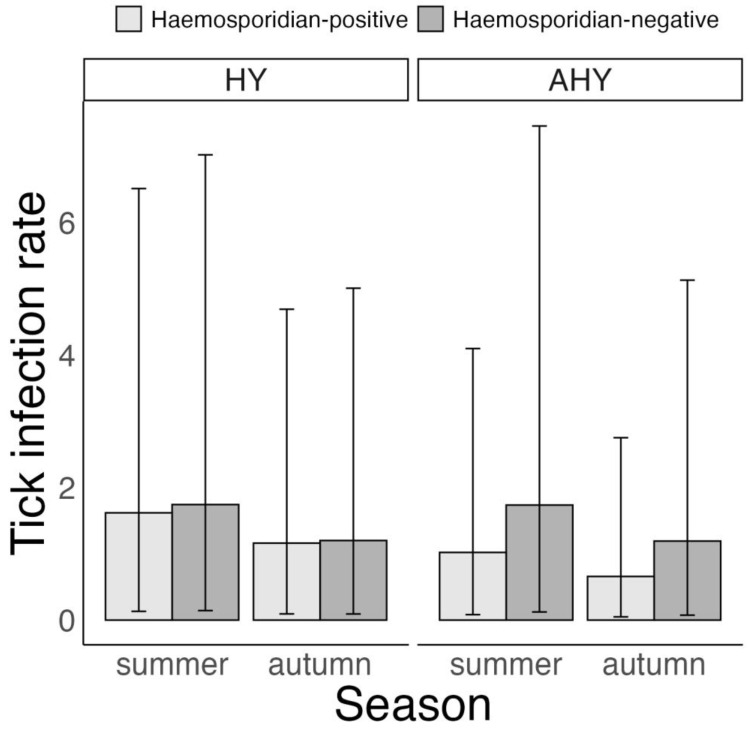
Bayesian zero inflated negative binomial model on variation in the rate of Borrelia-infected bird-feeding *Ixodes ricinus* ticks per host according to haemosporidian infection status of 4 avian host species in Slovakia, 2017–2019. The plotted rates are shown relative to the average tick infestation of 4.7 ticks per host. Displayed are posterior means and 95% CrI. Host age was classified as hatch-year (HY) and after-hatch-year (AHY). Note that spring was not considered in the model because only AHY birds were captured at that time of year.

Considering the summer–autumn period and assigning birds to HY and AHY age classes, the tick infection rate relative to the average infestation intensity (4.70 ticks), tended to be 0.4 ticks lower in haemosporidian-positive compared to haemosporidian-negative hosts (haemosporidian-positive vs. haemosporidian-negative hosts: –0.4 ticks (–1.36 to 0.16), *p* = 0.89). Importantly, this trend only holds for the group of AHY birds (haemosporidian-positive vs. haemosporidian-negative hosts—AHY birds: –0.63 ticks (–2.02 to 0), *p* = 0.95 and HY birds: –0.08 ticks (–0.54 to 0.28), *p* = 0.62, Figure 6 and Table S6).

## 4. Discussion

We studied whether the *Ixodes ricinus* tick infestation rates of birds as well as infection rates of bird-feeding *I. ricinus* ticks with bird-associated *Borrelia* spirochetes are linked to the hosts’ haemosporidian infection status. To our knowledge, this is the first study revealing the existence of such a link, namely, the negative association between the hosts’ haemosporidian infection status and the *Borrelia* infection rate of bird-feeding ticks.

The associations in parasite prevalences were studied from spring to autumn during three years for a community of woodland birds and their *I. ricinus* ectoparasites. Birds of 6 of the community’s 61 avian species harbored the majority of *I. ricinus* ticks. Both the tick infestation prevalence and intensity peaked during spring and summer, but while bird-feeding nymphs prevailed in spring, larvae dominated in summer. Almost half of the bird-feeding ticks were found positive for *B. burgdorferi* s.l. Even though the majority of infections involved bird-associated *B. garinii* and *B. valaisiana*, *B. garinii* appears to be the dominant *Borrelia* strain circulating in locally breeding avian species. First, *B. garinii* not only was more abundant than *B. valaisiana*, but it was relatively more prevalent in larvae than nymphs. Second, in summer, when numbers of all bird-feeding larvae as well as *Borrelia*-infected larvae peaked, *B. garinii* was relatively more prevalent than *B. valaisiana* in larvae. Third, unlike for *B. valaisiana*, *B. garinii* not only infected a more-than-expected number of larvae but also nymphs during the summer period. Finally, in contrast to *B. garinii*, *B. valaisiana* was rarely detected in bird-feeding larvae from avian hosts other than the Eurasian blackbird. Consequently, given the infrequent transovarial transmission of *B. burgdorferi* s.l., the infection pattern of *I. ricinus* ticks suggests that (1) *B. garinii* is readily transmitted at the study site by locally breeding birds, particularly by the Eurasian blackbird and song thrush, and (2) host-to-tick transmission of this spirochete locally takes place mainly in summer.

We considered tick- and host-centered scenarios to explain the variation in tick infestation rates and *Borrelia*-infection rates in bird-feeding ticks according to the haemosporidian infection status of avian tick hosts. Even though ticks were predicted in both scenarios to be generally more abundant on haemosporidian-positive bird hosts, our results reveal a complex system dependent on the host’s age and the time of year. In general, tick infestation prevalence and intensity were not clearly associated with the hosts’ haemosporidian infection status, except for autumn, when ticks were more prevalent and abundant on haemosporidian-negative hosts. Yet, closer examination of a subset of bird hosts of known age reveals that tick infestation intensity was higher for haemosporidian-positive birds, thereby supporting our prediction, but only for older (after-hatch-year, AHY) birds and only in summer. In turn, contrary to our prediction, tick infestation intensity for young (HY) birds showed a temporally consistent trend to be lower for haemosporidian-positive than haemosporidian-negative birds, though the probability of this difference exceeded 95% only for the period of autumn (*p* was exactly 95% for summer).

The results on tick infestation intensity for AHY birds during summer is particularly relevant in the context of our study system because summer coincides with both (1) the time of year with the highest haemosporidian parasitaemia [27] and, along with the period of spring, (2) the highest tick infestation intensity. In contrast, the result for HY birds and autumn is harder to interpret because haemosporidian parasitaemia [27] as well as tick infestation rates reach the lowest values in autumn. Consequently, while variation in tick infestation intensities among AHY birds is compatible with the idea that blood-feeding arthropods aggregate more on hosts with impaired immunity [42], the causes of the association between tick infestation intensity and the hosts’ haemosporidian infection status are pointing to other causes than impaired immunity for HY birds. It is feasible that haemosporidian-negative HY birds, particularly in autumn when tick ectoparasitism is low, largely include immunologically naïve hosts with low prior experience to ectoparasitism, thereby representing more susceptible hosts to blood-feeding ectoparasites than hosts with parasite-primed immunity [8,64,65,66]. In fact, HY birds consistently harbored, irrespective of their haemosporidian infection status, more ticks than AHY birds, even though this difference was weaker in summer. Altogether, our results on tick infestation intensity support the idea that immunologically more vulnerable hosts harbor more ticks, but suggest that different mechanisms may be responsible for infestation rates in immunologically naïve and experienced hosts. In addition, it is important to note that we did not detect the link between the hosts’ haemosporidian infection status and tick infestation intensity in spring (April) when only AHY birds were captured. While tick infestation intensity was high in spring, we found previously that haemosporidian infection intensity was low in this period [27]. Therefore, given that the host’s immune defense capacity is an important predictor of tick infestation intensity, our results suggest that host defense capacities against ticks do not vary greatly among AHY birds according to haemosporidian infection status during the pre-breeding period.

Our results on the association between *Borrelia* infection rates of bird-feeding ticks and haemosporidian infection of their avian hosts are consistent with the scenario in which *Borrelia* prevalences in bird-feeding ticks reflect the conflicting host defenses against extracellular and intracellular parasites [44]. Specifically, we found that the rates of ticks infected with bird-associated *Borrelia* spirochetes were lower if ticks fed on the hosts positive for avian haemosporidians. Therefore, this result is consistent with studies revealing that intracellular parasites, such as haemosporidians, can benefit from host immune systems prioritizing immune responses against extracellular parasites at the expense of immune responses against intracellular parasites [24,40,41,43]. The applicability of the hypothesis, based on the polarization of host immune responses, rests on the assumption that parasites eliciting conflicting immunological pathways are widespread in host populations. That is to say, this hypothesis assumes that lower rates of *Borrelia*-infected bird-feeding ticks result from the immune-induced reduction of host-to-tick transmission of *Borrelia* spirochetes, and not from the hosts being *Borrelia*-free. This assumption holds for our system, because avian haemosporidians and bird-associated *Borrelia* spirochetes are common parasites in the local avian community [26,27,67]. In fact, given the high contact rates of birds with ticks as well as high haemosporidian and *Borrelia* prevalences in birds and bird-feeding ticks, this and our previous study [27] indicate that for some bird species it can be virtually impossible to escape infection by bird-associated spirochetes and haemosporidians. Additionally, both haemosporidians and *Borrelia* spirochetes are thought to infect hosts early in life and persist in them for life as latent or recurrent infections [4,6,32,68]. Another assumption of the immune polarization hypothesis is that avian hosts should be able to mount immune responses to *Borrelia* spirochetes associated with such hosts. Indeed, contrary to earlier expectations based on the absence of clinical signs of *Borrelia* infection in avian hosts [69], the ability to mount host defenses against *Borrelia* spirochetes was confirmed for the key avian reservoir of bird-associated *Borrelia* spirochetes, the Eurasian blackbird [10,70,71].

Although our results on variation in *Borrelia* prevalences in bird-feeding ticks support the hypothesis based on the polarization of host immune responses, it is only in specific contexts. Namely, the hypothesis holds for (1) AHY hosts, (2) period of summer and autumn and only with respect to (3) the rate of *Borrelia*-infected ticks per host. There are several reasons why such context-dependence may be biologically and ecologically relevant for our study system. First, as indicated also by our results on tick infestation intensity, juvenile birds show poorly developed immune systems, including adaptive humoral responses, in the first months after fledging [72,73,74]. Therefore, the crucial immune response for regulating spirochetes, i.e., adaptive humoral responses [75], may not be sufficiently developed in HY birds in their first year of life, irrespective of their haemosporidian infection status. Second, while tick nymphs are thought to be the key transmitters of tick-borne parasites, larvae are supposed to function as parasite acquirers [76]. Our results show that nymphs prevail on birds in spring while larvae prevail in summer. Consequently, given the high infection rate of tick nymphs, bird hosts at our study site can readily be infected or reinfected by *Borrelia* during spring and transmit the spirochete to larvae in summer. Importantly, such an infection pattern would imply that AHY birds routinely face the acute phase of *Borrelia* infection in spring, whereas these birds can be in later phases of *Borrelia* infection with well-developed specific immune responses to regulate spirochetes in summer and autumn. Overall, summer appears to be ideal to detect host-related effects on *Borrelia* infection rates in bird-feeding ticks in our ecological contexts. Third, given the high *Borrelia* prevalences in bird-feeding ticks, the rate of infected ticks per host appears to be a more sensitive predictor of host effects on *Borrelia* tick-to-host and host-to-tick transmission rates than the probability that a host harbors at least one *Borrelia*-infected tick.

Overall, given the assumptions, our results are consistent with the idea that lower rates of *Borrelia*-infected ticks feeding on haemosporidian-positive avian hosts are the manifestation of host-polarized immune responses. We suggest the following scenario to take place in our system. In spring, the immunological response of AHY hosts to infestation by *Borrelia*-infected nymphs induces the pro-inflammatory T helper (Th)1-polarized immune pathway [75,77], resulting in the regulation of both extracellular *Borrelia* spirochetes and intracellular haemosporidians [78]. By summer, however, these hosts fight late spirochetosis mainly by means of adaptive humoral responses [75], switching their immune responses to the anti-inflammatory Th2-polarized pathway [77]. In this way, the hosts continue to regulate the spirochetes, but without risking Th1-induced immunopathology and at the cost of the hindered suppression of co-infecting intracellular apicomplexans [44,78]. Importantly, the lower rates of *Borrelia*-infected bird-feeding ticks in haemosporidian-positive birds may not only be due to reduced host-to-tick spirochete transmission, but also due to spirochete killing within bird-feeding ticks by ingested avian anti-*Borrelia* antibodies. In fact, such antibodies have recently been detected in the sera of Eurasian blackbirds [71], though an avian serum complement is thought to play the key role in *Borrelia* killing in ticks [71,79]. Further experimental research is needed to confirm our results and address the complexity of avian immunological mechanisms responsible for *Borrelia* suppression in hosts but also in feeding ticks. A recent work examined different extra- and intra-cellular parasites and different immune indices of Eurasian blackbirds, revealing that the associations between parasites and immune indices were strongly modulated by the only representative of extracellular parasites examined—intestinal cestodes [7]. As our results hold for multi-species data, not only for the Eurasian blackbird, it is possible that the immunological mechanisms based on the modulatory effect of extracellular parasites may be common across avian reservoirs.

Previously, three studies examined the associations between tick loads and/or tick infection with *Borrelia* spirochetes and haemosporidian infection in avian hosts [8,9,10]. None of the latter studies found a clear link between bird-feeding ticks, tick-borne *Borrelia* and haemosporidians, although Eurasian blackbirds infected with *Borrelia* tended to have a higher probability of haemosporidian infection in a captivity study [9]. Parasite associations were examined differently in our study compared to the previous two field studies. Specifically, while ticks, *Borrelia* spirochetes and haemosporidians were sampled throughout the year, temporal variability in tick, haemosporidian and host life cycles were not accounted for in previous studies [8,10]. The results of our study suggest that temporal changes in host–vector–parasite associations should be considered in future studies, because tick infestation and infection rates as well as host immune responses to these stimuli inevitably vary over time.

Host infection with extra-cellular spirochetes can result in the easier establishment and pathogenicity of intracellular apicomplexans [24,80]. Interestingly, previous studies revealed that the shift of Eurasian blackbirds to breed in urban environments can be related to the benefits accrued from lower tick infestation prevalence and intensity [81,82]. These authors suggested that Eurasian blackbirds breeding in urban habitats may achieve higher fitness due to the lower abundance of ticks and tick-borne parasites. Our study implies that birds associated with urban habitats may benefit not only from the lower risks of infection with tick-borne parasites, but also because their immune responses to intracellular parasites, such as haemosporidians, would not be hindered by infection with tick-borne extracellular parasites, such as *Borrelia* spirochetes. The alleviation of haemosporidian infection in birds of urban habitats can be particularly beneficial for Eurasian blackbirds, because haemoproteids associated with this bird are known to be highly pathogenic [83,84]. In fact, Eurasian blackbirds across European urban areas show both lower tick and haemosporidian prevalences [81]. As the community of parasites of birds such as the Eurasian blackbird is rich [7,10], this study supports the claim that a more robust design of parasitological studies is needed to understand the ecology of interactions among hosts and their parasites as well as the evolutionary causes of changes in parasite pathogenicity or the species–habitat associations of avian hosts [85].

## Figures and Tables

**Table 1:** Prevalence of *Borrelia burgdorferi* s.l. in *Ixodes ricinus* ticks feeding on birds in Slovakia, 2017–2019. L and N refer to larvae and nymphs, respectively. Restriction Fragment Length Polymorphism (RFLP) was used to diagnose the genospecies of borreliae.

Bird Species	No. Birds Sampled	No. Birds Infested with Ticks	No. Birds with Infected Ticks	No. Ticks	No. *B. burgdorferi* s.l. Infected Ticks	No. *B. garinii* Infected Ticks (L/N)	No. *B. valaisiana* Infected Ticks (L/N)	No. *B. garinii* + *B. valaisiana* Infected Ticks (L/N)	No. *B. afzelii* Infected Ticks (L/N)	No. *B. lusitaniae* Infected Ticks (L/N)	No. *B. burgdorferi* s.s Infected Ticks (L/N)	No. *B. spielmanii* Infected Ticks (L/N)	No. *B. afzelii* + *B. garinii* Infected Ticks (L/N)	No. *B. garinii* + *B. lusitaniae* Infected Ticks (L/N)	No. *B. afzelii* + *B. valaisiana* Infected Ticks (L/N)	No. *B. b.* s.s. + *B. lusitaniae* Infected Ticks (L/N)	No. *B. garinii* + ? Infected Ticks (L/N)	No. *B. valaisiana* + ? Infected Ticks (L/N)
*C. coccothraustes*	44	19	5	60	7	1 (0/1)	3 (0/3)	1 (0/1)	2 (1/1)	0	0	0	0	0	0	0	0	0
*Cyanistes caeruleus*	121	2	0	3	0	0	0	0	0	0	0	0	0	0	0	0	0	0
*Emberiza schoeniclus*	3	1	0	1	0	0	0	0	0	0	0	0	0	0	0	0	0	0
*Erithacus rubecula*	355	120	21	338	26	12 (8/4)	7 (1/6)	0	6 (0/6)	0	0	1 (1/0)	0	0	0	0	0	0
*Fringilla coelebs*	31	4	3	16	5	2 (0/2)	0	0	3 (0/3)	0	0	0	0	0	0	0	0	0
*Garrulus glandarius*	11	5	0	20	0	0	0	0	0	0	0	0	0	0	0	0	0	0
*Chloris chloris*	12	2	1	2	1	0	0	0	1 (0/1)	0	0	0	0	0	0	0	0	0
*Luscinia megarhynchos*	17	5	0	9	0	0	0	0	0	0	0	0	0	0	0	0	0	0
*Parus major*	175	35	8	67	8	4 (3/1)	2 (1/1)	0	2 (0/2)	0	0	0	0	0	0	0	0	0
*Passer montanus*	5	1	0	1	0	0	0	0	0	0	0	0	0	0	0	0	0	0
*Phylloscopus collybita*	74	3	0	4	0	0	0	0	0	0	0	0	0	0	0	0	0	0
*Phylloscopus trochilus*	27	2	0	2	0	0	0	0	0	0	0	0	0	0	0	0	0	0
*Poecile palustris*	23	1	0	3	0	0	0	0	0	0	0	0	0	0	0	0	0	0
*Prunella modularis*	75	1	1	1	1	0	0	0	1 (0/1)	0	0	0	0	0	0	0	0	0
*Pyrrhula pyrrhula*	39	5	1	9	1	0	0	0	1 (0/1)	0	0	0	0	0	0	0	0	0
*Sylvia atricapilla*	372	17	0	27	0	0	0	0	0	0	0	0	0	0	0	0	0	0
*Sylvia communis*	12	3	0	9	0	0	0	0	0	0	0	0	0	0	0	0	0	0
*T. troglodytes*	29	1	0	3	0	0	0	0	0	0	0	0	0	0	0	0	0	0
*Turdus merula*	96	86	76	909	610	322 (204/118)	179 (57/122)	81 (29/52)	11 (0/11)	4 (0/4)	3 (0/3)	0	3 (0/3)	1 (0/1)	1 (0/1)	1 (0/1)	1 (0/1)	3 (1/2)
*Turdus philomelos*	40	27	19	169	105	86 (63/23)	6 (0/6)	10 (5/5)	2 (0/2)	0	0	0	0	1 (0/1)	0	0	0	0
Total	1561	340	135	1653	764	427 (278/148)	197 (59/138)	92 (34/58)	29 (1/28)	4 (0/4)	3 (0/3)	1 (1/0)	3 (0/3)	2 (0/2)	1 (0/1)	1 (0/1)	1 (0/1)	3 (1/2)

## Data Availability

Not applicable.

## Supplementary Materials

The following supporting information can be downloaded at: https://www.mdpi.com/article/10.3390/microorganisms11010060/s1, Table S1: Bayesian Bernoulli distribution model on variation in *I. ricinus* infestation probability; Table S2: Bayesian zero inflated negative binomial model on variation in *I. ricinus* infestation intensity; Table S3: Bayesian zero inflated negative binomial model on variation in *I. ricinus* tick infestation intensity; Table S4: Bayesian Bernoulli distribution model on variation in the probability of *Borrelia*-infection of bird-feeding *I. ricinus* ticks; Table S5: Bayesian zero inflated negative binomial model on variation in the proportion of *Borrelia*-infected bird-feeding *I. ricinus* ticks; Table S6: Bayesian zero inflated negative binomial model on variation in the rate of *Borrelia*-infected bird-feeding *I. ricinus* ticks; Figure S1: Variation in *I. ricinus* tick infection intensity.
