# Supplementary material for: The Infection Rate of Bird-Feeding Ixodes ricinus Ticks with Borrelia garinii and B. valaisiana Varies with Host Haemosporidian Infection Status
Source: Microorganisms. 2022 Dec 25;11(1):60. doi: 10.3390/microorganisms11010060 (PMC9861293; doi:10.3390/microorganisms11010060)
Supplement: Supplementary file 1 [file microorganisms-11-00060-s001.zip › microorganisms-2104572-supplementary.pdf]

Table S1. Bayesian Bernoulli distribution model on variation in *Ixodes ricinus* infestation probability according to haemosporidian infection status of six avian host species in Slovakia, 2017–2019. Posterior estimates are based on data involving 1040 birds of Eurasian blackbird *Turdus merula*, song thrush *Turdus philomelos*, European robin *Erithacus rubecula*, hawfinch *C. coccothraustes*, great tit *Parus major*, and Eurasian blackcap *Sylvia atricapilla*. The model was fitted using four independent Markov chains, each with 20,000 iterations and 500 warm-up samples.

Model parameters	Estimate	Est. Error	l-95 % CrI	u-95 % CrI	R-hat	Bulk ESS	Tail ESS
Group-level effects:							
Year (3 levels)							
SD (Intercept)	1.05	1.03	0.03	3.68	1.00	1588	571
Host species nested in Year (18 levels)							
SD (Intercept)	2.49	0.52	1.67	3.71	1.00	12732	34050
Population-level effects:							
Intercept	-1.41	0.88	-3.18	0.36	1.00	3605	2337
Haemosporidian infection status (negative) [HIS_n]	0.65	0.30	0.08	1.25	1.00	9014	13758
Time of year (spring) [TY_spring]	1.99	0.37	1.27	2.74	1.00	12624	14852
Time of year (summer) [TY_summer]	1.38	0.36	0.69	2.09	1.00	10909	36995
HIS_n × TY_spring	-1.02	0.48	-1.97	-0.10	1.00	4920	31484
HIS_n × TY_summer	-0.58	0.43	-1.43	0.26	1.00	19257	35243

Table S2. Bayesian zero inflated negative binomial model on variation in *Ixodes ricinus* infestation intensity according to haemosporidian infection status of six avian host species in Slovakia, 2017–2019. Posterior estimates are based on data involving 1040 birds of Eurasian blackbird *Turdus merula*, song thrush *Turdus philomelos*, European robin *Erithacus rubecula*, hawfinch *C. coccothraustes*, great tit *Parus major*, and Eurasian blackcap *Sylvia atricapilla*. The model was fitted using four independent Markov chains, each with 20,000 iterations and 500 warm-up samples.

Model parameters	Estimate	Est. Error	l-95 % CrI	u-95 % CrI	R-hat	Bulk ESS	Tail ESS
Group-level effects:							
Year (3 levels)							
SD (Intercept)	0.90	0.83	0.03	3.08	1.01	623	339
Host species nested in Year (18 levels)							
SD (Intercept)	2.02	0.40	1.40	2.92	1.01	1467	9614
Population-level effects:							
Intercept	-1.42	0.75	-3.00	0.12	1.01	676	414
Haemosporidian infection status (negative) [HIS_n]	0.64	0.23	0.19	1.07	1.00	1691	5344
Time of year (spring) [TY_spring]	1.65	0.24	1.18	2.13	1.00	3177	3760
Time of year (summer) [TY_summer]	1.56	0.23	1.11	2.01	1.00	3440	11769
HIS_n × TY_spring	-0.63	0.33	-1.28	0.03	1.00	4125	2141
HIS_n × TY_summer	-0.56	0.29	-1.13	0.02	1.00	3168	5083
Family specific parameters							
Shape	0.69	0.11	0.52	0.95	1.00	1475	4960
zi	0.06	0.05	0.00	0.17	1.00	1777	15273

Table S3. Bayesian zero inflated negative binomial model on variation in *Ixodes ricinus* tick infestation intensity according to haemosporidian infection status and age of six avian host species in Slovakia, 2017–2019. Posterior estimates are based on data involving 749 birds of Eurasian blackbird *Turdus merula*, song thrush *Turdus philomelos*, European robin *Erithacus rubecula*, hawfinch *C. coccothraustes*, great tit *Parus major*, and Eurasian blackcap *Sylvia atricapilla*. The model was fitted using four independent Markov chains, each with 20,000 iterations and 500 warm-up samples. Host age was classified as hatch-year (HY) and after-hatch-year (AHY).

Model parameters	Estimate	Est. Error	l-95 % CrI	u-95 % CrI	R-hat	Bulk ESS	Tail ESS
Group-level effects:							
Year (3 levels)							
SD (Intercept)	0.82	0.78	0.02	2.92	1.00	3036	2403
Host species nested in Year (18 levels)							
SD (Intercept)	1.84	0.37	1.26	2.70	1.00	5772	12200
Population-level effects:							
Intercept	-1.53	0.79	-3.24	-0.03	1.00	1966	827
Haemosporidian infection status (positive) [HIS_p]	0.20	0.48	-0.74	1.13	1.00	10673	35285
Time of year (summer) [TY_summer]	0.99	0.53	-0.03	2.04	1.00	2542	1294
Age (HY) [A_HY]	0.96	0.40	0.17	1.75	1.00	5512	7609
HIS_p × TY_summer	0.69	0.69	-0.64	2.07	1.00	3231	4578
HIS_p × A_HY	-1.05	0.54	-2.10	0.01	1.00	7997	21424
TY_summer × A_HY	0.00	0.57	-1.13	1.11	1.00	2522	1228
HIS_p × TY_summer × A_HY	-0.29	0.78	-1.83	1.25	1.00	3236	1501
Family specific parameters							
Shape	0.68	0.14	0.47	1.02	1.00	11984	35261
zi	0.08	0.06	0.00	0.22	1.00	4164	1400

Table S4. Bayesian Bernoulli distribution model on variation in the probability of *Borrelia*-infection of bird-feeding *Ixodes ricinus* ticks per host according to haemosporidian infection status of five avian host species in Slovakia, 2017–2019. Posterior estimates are based on data involving 685 birds of Eurasian blackbird *Turdus merula*, song thrush *Turdus philomelos*, European robin *Erithacus rubecula*, hawfinch *C. coccothraustes*, and great tit *Parus major*. The model was fitted using four independent Markov chains, each with 20,000 iterations and 500 warm-up samples.

Model parameters	Estimate	Est. Error	l-95 % CrI	u-95 % CrI	R-hat	Bulk ESS	Tail ESS
Group-level effects:							
Year (3 levels)							
SD (Intercept)	1.20	1.07	0.04	3.99	1.00	2229	1054
Host species nested in Year (15 levels)							
SD (Intercept)	2.49	0.58	1.61	3.85	1.00	10782	12800
Population-level effects:							
Intercept	-2.37	1.06	-4.50	-0.12	1.00	2252	1173
Haemosporidian infection status (positive) [HIS_p]	-0.19	0.51	-1.20	0.82	1.00	21076	39269
Time of year (spring) [TY_spring]	1.44	0.66	0.11	2.73	1.00	13210	9808
Time of year (summer) [TY_summer]	1.15	0.51	0.16	2.16	1.00	16379	18826
HIS_p × TY_spring	0.35	0.82	-1.26	1.98	1.00	14857	26455
HIS_p × TY_summer	0.62	0.70	-0.73	2.01	1.00	15786	37957

Table S5. Bayesian zero inflated negative binomial model on variation in the proportion of *Borrelia*-infected bird-feeding *Ixodes ricinus* ticks per host according to haemosporidian infection status of four avian host species in Slovakia, 2017–2019. The rate was calculated using host infestation by ticks as the offset parameter. Posterior estimates are based on data involving 261 birds of Eurasian blackbird *Turdus merula*, song thrush *Turdus philomelos*, European robin *Erithacus rubecula*, and great tit *Parus major*. The model was fitted using four independent Markov chains, each with 20,000 iterations and 500 warm-up samples.

Model parameters	Estimate	Est. Error	l-95 % CrI	u-95 % CrI	R-hat	Bulk ESS	Tail ESS
Group-level effects:							
Year (3 levels)							
SD (Intercept)	0.19	0.34	0.00	1.10	1.00	17330	20212
Host species (4 levels)							
SD (Intercept)	1.70	0.79	0.73	3.77	1.00	18691	14238
Population-level effects:							
Intercept	-1.95	0.90	-3.89	-0.23	1.00	21682	23516
Haemosporidian infection status (positive) [HIS_p]	0.03	0.27	-0.49	0.58	1.00	26324	34980
Time of year (spring) [TY_spring]	-0.10	0.27	-0.61	0.44	1.00	28957	37622
Time of year (summer) [TY_summer]	0.58	0.26	0.08	1.10	1.00	28117	34997
HIS_p × TY_spring	0.21	0.31	-0.41	0.82	1.00	27248	37309
HIS_p × TY_summer	-0.24	0.30	-0.83	0.34	1.00	26935	35016
Family specific parameters							
Shape	86.74	69.10	18.29	276.57	1.00	64823	51479
zi	0.07	0.03	0.02	0.14	1.00	63760	35346

Table S6. Bayesian zero inflated negative binomial model on variation in the rate of *Borrelia*-infected bird-feeding *Ixodes ricinus* ticks per host according to haemosporidian infection status and age of four avian host species in Slovakia, 2017–2019. The rate was calculated using host infestation by ticks as the offset parameter. Posterior estimates are based on data involving 197 birds of Eurasian blackbird *Turdus merula*, song thrush *Turdus philomelos*, European robin *Erithacus rubecula*, and great tit *Parus major*. The model was fitted using four independent Markov chains, each with 20,000 iterations and 500 warm-up samples. Host age was classified as hatch-year (HY) and after-hatch-year (AHY).

Model parameters	Estimate	Est. Error	l-95 % CrI	u-95 % CrI	R-hat	Bulk ESS	Tail ESS
Group-level effects:							
Year (3 levels)							
SD (Intercept)	0.29	0.54	0.01	1.62	1.00	5447	2355
Host species (4 levels)							
SD (Intercept)	1.80	0.82	0.78	3.95	1.00	23990	24098
Population-level effects:							
Intercept	-1.80	0.97	-3.85	0.15	1.00	8173	2926
Haemosporidian infection status (positive) [HIS_p]	0.00	0.34	-0.64	0.68	1.00	26415	38147
Time of year (summer) [TY_summer]	0.41	0.32	-0.19	1.05	1.00	28413	41062
Age (AHY) [A_AHY]	-0.06	0.57	-1.20	1.02	1.00	24477	37472
HIS_p × TY_summer	-0.08	0.36	-0.80	0.60	1.00	25561	37990
HIS_p × A_AHY	-0.55	0.66	-1.81	0.76	1.00	22109	38042
TY_summer × A_AHY	0.00	0.69	-1.34	1.35	1.00	25064	36342
HIS_p × TY_summer × A_AHY	0.14	0.78	-1.40	1.66	1.00	22385	34115
Family specific parameters							
Shape	116.26	84.16	24.04	337.77	1.00	63163	55223
zi	0.08	0.04	0.02	0.17	1.00	55899	33165

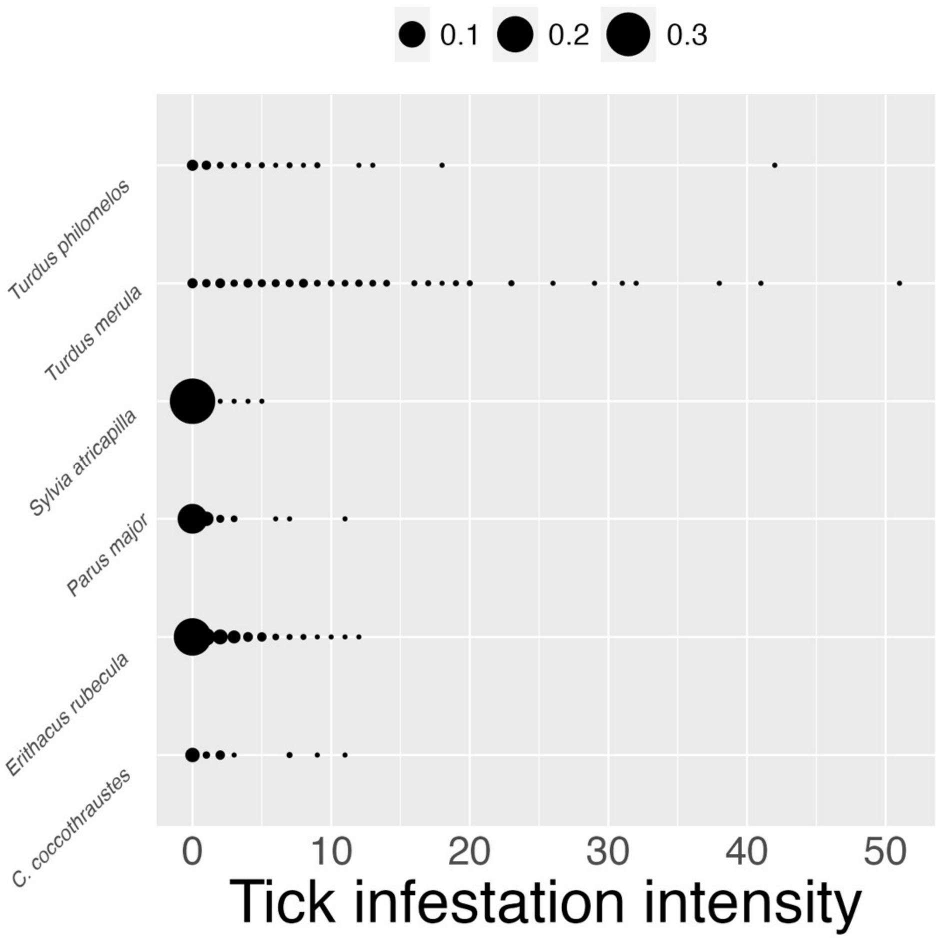

Figure S1. Variation in Ixodes ricinus infection intensity in six avian host species in Slovakia, 2017–2019. Tick infestation intensity is based on data involving 1082 birds of Eurasian blackbird Turdus merula (n=96), song thrush Turdus philomelos (n=40), European robin Erithacus rubecula (n=355), hawfinch C. coccothraustes (n=44), great tit Parus major (n=175), and Eurasian blackcap Sylvia atricapilla (n=372). The size of circle refers to the proportion of cases observed for a given tick infestation intensity level. Note that these samples include one European robin with tick (infestation and Borrelia-infection) data but absent blood sample.
